# Rotating Hinge Knee Arthroplasty for Revision Prosthetic-Knee Infection: Good Functional Outcomes but a Crucial Need for Superinfection Prevention

**DOI:** 10.3389/fsurg.2021.551814

**Published:** 2021-09-20

**Authors:** Florian Bourbotte-Salmon, Tristan Ferry, Mickaël Cardinale, Elvire Servien, Frédéric Rongieras, Michel-Henry Fessy, Antoine Bertani, Frédéric Laurent, Margaux Buffe-Lidove, Cécile Batailler, Sébastien Lustig, The Lyon Bone and Joint Infections Study Group

**Affiliations:** ^1^Department of Orthopaedic and Traumatologic Surgery, Hôpital Edouard Herriot, Lyon, France; ^2^Service des Maladies Infectieuses et Tropicales, Hôpital de la Croix-Rousse, Hospices Civils de Lyon, Lyon, France; ^3^Université Claude Bernard Lyon 1, Lyon, France; ^4^Centre interrégional de Référence pour la prise en charge des Infections Ostéo-Articulaires complexes (CRIOAc Lyon), Hospices Civils de Lyon, Lyon, France; ^5^CIRI – Centre International de Recherche en Infectiologie, Inserm, U1111, Université Claude Bernard Lyon 1, CNRS, UMR5308, Ecole Normale Supérieure de Lyon, Univ Lyon, Lyon, France; ^6^Department of Anesthesiology and Intensive Care, Hôpital d'Instruction des Armées Saint-Anne, Toulon, France; ^7^Department of Orthopaedic and Sport Surgery, Hôpital de la Croix Rousse, Lyon, France; ^8^Department of Orthopaedic and Traumatologic surgery, Centre Hospitalier Lyon Sud, Pierre-Bénite, France; ^9^Institut des Agents Infectieux, Hôpital de la Croix Rousse, Lyon, France; ^10^Department of Physical and Rehabilitation Medicine, Hôpital d'Instruction des Armées Desgenettes, Lyon, France

**Keywords:** arthroplasty, total knee arthroplasty, knee prosthesis, prosthetic-joint infection, septic revision, superinfection, prevention

## Abstract

**Introduction:** Management of chronic infection following total knee arthroplasty (TKA) is challenging. Rotating hinged prostheses are often required in this setting due to severe bone loss, ligamentous insufficiency, or a combination of the two. The nature of the mechanical and septic complications occurring in this setting has not been well-described. The aim of this study was to evaluate patient outcomes using a hinge knee prosthesis for prosthetic knee infections and to investigate risk factors for implant removal.

**Methods:** This was a retrospective cohort study that included all patients treated in our tertiary level referral center between January 2009 and December 2016 for prosthetic knee infection with a hinge knee prosthesis. Only patients with a minimum 2-year of follow-up were included. Functional evaluation was performed using international knee society (IKS) “Knee” and “Function” scores. Survival analysis comparing implant removal risks for mechanical and septic causes was performed using Cox univariate analysis and Kaplan-Meier curves. Risk factors for implant removal and septic failure were assessed.

**Results:** Forty-six knees were eligible for inclusion. The majority of patients had satisfactory functional outcomes as determined by mean IKS scores (mean knee score: 70.53, mean function score: 46.53 points, and mean knee flexion: 88.75°). The 2-year implant survival rate was 89% but dropped to 65% at 7 years follow-up. The risk of failure (i.e., implant removal) was higher for septic etiology compared to mechanical causes. Patients with American society of anesthesiologists (ASA) score>1, immunosuppression, or with peripheral arterial diseases had a higher risk for septic failure. Patients with acute infection according to the Tsukayamaclassification had a higher risk of failure. Of the 46 patients included, 19 (41.3%) had atleast one infectious event on the surgical knee and most of these were superinfections (14/19) with new pathogens isolated. Among pathogens responsible for superinfections (i) cefazolin and gentamicin were both active in six of the cases but failed to prevent the superinfection; (ii) cefazolin and/or gentamicin were not active in eight patients, leading to alternative systemic and/or local antimicrobial prophylaxis consideration.

**Conclusions:** Patients with chronic total knee arthroplasty (TKA) infection, requiring revision using rotating hinge implant, had good functional outcomes but experienced a high rate of septic failure, mostly due to bacterial superinfection. These patients may need optimal antimicrobial systemic prophylaxis and innovative approaches to reduce the rate of superinfection.

## Introduction

Prosthetic-joint infection (PJI) is a devastating complication after total knee arthroplasty (TKA). The rate of PJI following primary TKA is ~1–2% (1–4). The rate of bacterial resistance or a *de novo* infection (also called superinfection) is significantly higher in patients with chronic infection requiring prosthesis revision. Management is challenging, requiring a multi-disciplinary approach to determine the optimal strategy for prosthesis choice (non-constrained or constrained), staging surgery or not (single vs. two stage), the duration and delivery of systemic antimicrobial therapy, and the choice of antimicrobial prophylaxis at the time of reimplantation.

Hinged knee prostheses are often used in the revision of TKA (5). The indications for a hinged TKA are restricted to limb-salvage procedures such as tumor, complex fracture, or revision surgery with significant bone loss or collateral ligaments failure (6–11). In limited situations, the hinged knee prosthesis may be indicated in a primary setting, such as severe deformity (12).

The longevity of hinged TKAs remains a major concern, with high rates of mechanical complications being widely reported (8, 13–15). In order to limit such complications, prosthesis design has evolved and the third generation of rotating hinged TKA (RHTKA) has been available since 1999 (16, 17). The addition of a rotating platform allows increased freedom of movement compared to previous designs with the rationale of reducing force transmission at the implant-cement-bone interface. This implant could be used in the revision setting for the treatment of infected TKA, but data regarding outcomes when used for this indication remain limited and are heterogeneous (18–20).

Since 2009, third-generation rotating hinge knee prosthesis has been used in our institution for septic TKA revision surgery. The purpose of this study was to analyze the outcomes of patients with the use of this prosthesis for septic TKA revisions and to determine risk factors for mechanical and septic failures.

## Materials and Methods

This retrospective study was conducted at our regional referral center for the management of complex bone and joint infection called CRIOAc Lyon (http://www.crioac-lyon.fr). Patients who underwent RHTKA for septic revisions from 2009 to 2016 were included. This study received local institutional ethics approval. Patients were selected from the Lyon BJI cohort study (NCT02817711), and a dedicated data collection was performed for this study (NCT02856971).

### Diagnostic Criteria for TKA Infection

The diagnosis was made using the criteria of TKA infection according to the International Consensus Meeting on Prosthetic Joint Infections (21). Prosthetic joint infection was classified according to the Tsukayama and Zimmerli classifications that have been well-described (2, 22).

### Therapeutic Strategy

All prosthetic infections are discussed at a weekly multidisciplinary meeting. Our institution was responsible for the recommended prophylaxis guidelines included in the WHO surgical site infection (SSI) prevention recommendations (23). Cephazolin was routinely used for antimicrobial prophylaxis (in addition to the antimicrobial therapy used to treat the current infection) during prosthesis removal and reimplantation, according to national guidelines (24). For revision surgery, the scar was routinely excised and a trans-quadricipital tendon approach was used for arthrotomy. Additional exposure was achieved with an anterior tibial tuberosity (ATT) osteotomy, if required (*n* = 7). A 4.5 mm hole was drilled in the anterior cortex of both femur and tibia to mark the joint line for later reconstruction (25). Well-fixed prostheses were removed with a combination of sharp osteotomes, and cement was removed with the OSCAR® system (Orthosonics, Edimburg, United Kingdom). Numerous surgical samples were taken before administering antimicrobials (approved during the multi-disciplinary meeting), and seven samples were taken for bacteriological analysis and one for pathology. Then, extensive debridement and synovectomy were made, including the posterior cruciate ligament if there was any remaining stump after removal of implants. Pulsed lavage irrigation of the joint was performed with at least 6 L of saline solution. In patients for whom a 2-stage procedure was proposed, a gentamicin-loaded cement spacer was implemented with PALACOS® R+G (high viscosity), containing 0.8 g of gentamicin per 40 g of cement. The spacer was either articulating or static, depending on the condition of the local bone and soft tissues. ATT osteotomies were stabilized with non-resorbable transosseous sutures. The wound was closed with drainage left in place for 3 days. Patients wore a molded resin cast after implant removal. Patients were made strictly non-weight bearing until the second-stage surgery. Intensive physiotherapy began the day after the surgery, based on gait rehabilitation with walking aids. The second-stage surgery (reimplantation) was scheduled in patients with favorable local conditions and for whom the infection was deemed to be controlled. It was carried out under antibiotics or after an antibiotic window depending on the time since the explantation.

For the second-stage surgery, a large synovectomy was repeated. Collateral ligaments were dissected but not excised. Bone defects were managed either with bone cement or with wedges. All reimplanted hinged TKAs were fixed with high viscosity gentamicin-loaded cement (PALACOS® R+G). ATT osteotomies were secured with two cortical screws. The drainage was removed the day after the surgery. Physiotherapy started on the first post-operative day. Full weight bearing was allowed for single-stage exchange patients. Bacterial cultures were performed, and antibiograms were generated for all cultured bacteria. The antibiotic prescription was managed by infectious disease specialists during multidisciplinary meetings, with empirical antimicrobial therapy (no fixed protocol), and then targeted antimicrobial therapy prescribed according to the French and international guidelines. A total course of 3 months of antimicrobial therapy is the standard period of systemic therapy in our institution.

### Outcome Assessment

The aim of the study was to evaluate patient outcomes and implant survival. We evaluated the survival rates of rotating hinge knee prosthesis by comparing the risk for failure (i.e., implant removal) due to mechanical vs. septic causes, using a Cox univariate analysis (Hazard ratio, HR; 95% confidence interval, CI) and Kaplan-Meier curves (Log-rank test) (26). Risk factors for prosthesis removal were identified regardless of the cause. Patients with septic failure were additionally assessed with antibiograms of the strains responsible for the relapse to determine sensitivity to cefazolin (used as systemic antimicrobial prophylaxis) and gentamicin (used as local antimicrobial prophylaxis in the cement). Finally, risk factors for septic failure (i.e., need for subsequent surgery such as Debridement Antibiotics and Implant Retention [DAIR] or implant removal due to clinical signs of infection occurrence) were specifically evaluated with univariate Cox analysis. Risk factors for infectious events were analyzed using the following items: “age,” “ASA score > 1,” “immunosuppression,” “acute infection as initial clinical presentation according to Tsukayama classification,” “acute infection as initial clinical presentation according to Zimmerli classification,” “peripheral arterial disease.” IKS ≪ knee ≫ and ≪ function ≫ scores (International Knee Surgery) (27) were calculated for all patients who still had their prostheses at the last medical examination.

### Statistical Analysis

Multivariate Cox analyses were performed using the most significant determinants (*p* < 0.05) identified in the univariate analysis with another determinant. Due to the low sample size of the population, we did not include >2 variables into a single multivariate model. A *p*-value < 0.05 was considered significant. Statistical analyses were performed using SPPS Statistics Base 17.0 (Softonic International, San Francisco, CA, USA). Percentages of patients with or without characteristics of interest were compared using chi-square or Fisher's exact test, as appropriate.

## Results

During the study period, 230 patients were treated in our institution for infected TKAs. The indications for hinged TKAs used are presented in Table 1. Patients who underwent a revision of septic TKA by any other type of prosthesis than hinged prostheses (*n* = 180) and patients who underwent TKA revision with a hinged prosthesis for mechanical problems (*n* = 35) were excluded. Fifty patients who underwent revisions with hinged TKA for septic revision were eligible for inclusion. The population characteristics are presented in Table 2. Four patients were lost to follow-up before 24 months, including one patient who died after the revision (prostatic cancer). Another patient died after 2 years of follow-up (pulmonary embolism). This patient was included in the analysis and considered as lost to follow-up at the date of death. The number of hinged TKAs followed over 2 years was, therefore, 46, with a mean follow-up of 38.1 months [10; 88].

**Table 1:** Main indications of the use of hinged total knee arthroplasty (TKA) (*n* = 50 knees).

**Indication**	***n*** **(%)**
Hinged TKA revision	15 (30%)
Collateral ligaments deficiency	12 (24%)
Bone losses (AORI III)	15 (30%)
Femur	8 (16%)
Tibia	3 (6%)
Femur + Tibia	4 (8%)
Patella baja with ATT osteotomy required	6 (12%)
Complex periprosthetic open fracture	2 (4%)

**Table 2:** Population characteristics (50 patients).

**Item**	
Males (*n*, %)	22 (44)
Females (*n*, %)	28 (56)
Mean age^a^ in years (± SD)	73.04 ± 10.19
Medical history / risk factors for infection related to the host (n, %)	
- TKA previous infection	17 (34)
- Immunosuppression^b^	10 (20)
- Diabetes	16 (32)
- Rheumatoid arthritis	5 (10)
- Pre-operative anticoagulant	15 (30)
- Cirrhosis	1 (2)
- Antecedent of surgery on the index knee	22 (44)
Mean ASA^c^ score	2.36
Mean number of surgeries before the index TKA^d^(±SD)	0.87 ± 1.56
Mean number of surgeries before the hinged TKA^d^ (±SD)	5.04 ± 2.47
Type of infection (n, %)	
- Early infection <1 month	17 (34)
- Sub-acute infection <3 months	4 (8)
- Chronic infection	22 (44)
- Acute hematogenous infection	5 (10)
- Unknown	2 (4)

a *Mean age at the time of the hinged TKA implantation*.

b *Immunosuppression: any cause except diabetes, including long-term corticosteroids intake, Rheumatoid arthritis with cortioids and/or Methotrexate, cirrhosis, malignant hemopathy, chronic renal failure with cockroft <30 μmol/mL, solid cancer with immunomodulators, or chemotherapy*.

c *Physical status score of the American Society of Anaesthesiologists (ASA)*.

d *Any surgery including arthroscopies*.

Out of the 46 patients, 43 (93.5%) were managed with two-stage revision surgery. A cement spacer was used in 40 cases (static, *n* = 13; articulated, *n* = 27), and 3 patients were not given a spacer during the implant removal surgery because soft tissues did not allow. The average time between implant removal and second-stage reimplantation was 9.3 weeks. Thirty-six patients (81.2%) underwent second-stage reimplantation before 12 weeks, and most (*n* = 28) were reimplanted with adequate antimicrobial treatment. In these latter patients, the average time between implant removal and second-stage reimplantation was 8.9 weeks. Among the patients for whom a two-stage approach was performed, an antibiotic window before reimplantation was planned in 15/43 patients (34.9%) with an average time between implant removal and the reimplantation of 10.1 weeks.

Patients were selected for single-staged exchange (*n* = 4) if they had severe co-morbidities rendering an unfavorable risk-benefit ratio from two-stage management. One patient had a prosthetic loosening for which the septic origin was not suspected, until the results of intraoperative bacteriological samples returned positive.

**Figure 1:**
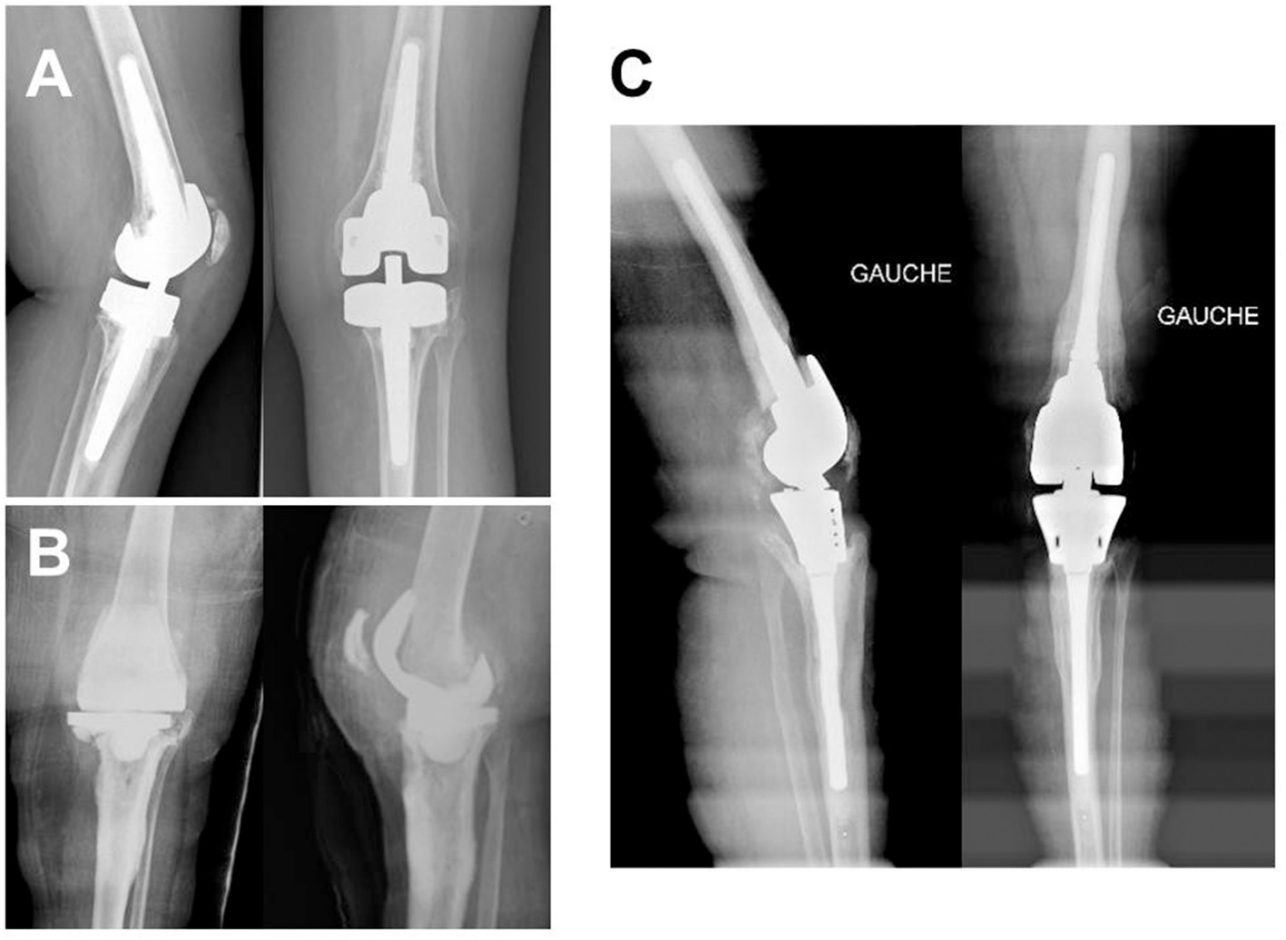
Typical x-ray from a patient with total knee arthroplasty (TKA) infection treated with a two-stage approach with reimplantation of a cemented hinged TKA: An 80-year-old female patient with a history of *Staphylococcus caprae* TKA infection **(A)** from whom explantation was performed (**B**, a gentamicin-cement spacer was used to fill the gap) and for whom reimplantation of a gentamicin-cemented hinged prosthesis because of important femoral and tibial bone loss AORI III **(C)**. The outcome was favorable at 3 years of follow-up.

The rotating hinged prostheses used are presented in Table 3. The distribution of the pathogens responsible for the initial TKA infection is presented in Table 4. No organism was found in five patients, who nevertheless met the described TKA infection criteria. Concerning patients with a two-stage exchange, a “second look” surgery (spacer exchange) was performed in 5/43 (11.6%) cases before reimplantation. Six patients (13.1%) benefited from at least one plastic surgery procedure for soft tissue losses before reimplantation. A typical x-ray of a patient with a hinged prosthesis used for revision is described in Figure 1.

**Table 3:** Hinged prostheses used (50 patients).

**Prothesis**	***n*** **(%)**
OSS^TM^ RHK^a^ (Biomet Zimmer®)	32 (64)
AXEL II (BBraun®)	13 (26)
LEXA (C2F®)	4 (8)
ROTAX (Lépine®)	1 (2)
Distal femoral replacement	12 (24)
Proximal tibial replacement	3 (6)
Both distal femoral and proximal tibial replacement	4 (8)
“Standard” Hinged TKA	31 (62)

a *Rotating hinge knee*.

**Table 4:** Distribution of the pathogens responsible for index TKA infections (50 patients).

**Pathogens**	***n*** **(%)**
*Staphylococcus*	15 (32.6)
Methicillin-susceptible *S. aureus*	4 (8.7)
Methicillin-resistant *S. aureus*	1 (2.3)
Methicillin-susceptible CNS^a^	5 (10.8)
Methicillin-resistant CNS^a^	5 (10.8)
*Streptococcus* spp.	10 (21.7)
*Cutibacterium acnes*	4 (8.7)
Gram-negative bacilli	3 (6.6)
Polymicrobial	9 (19.6)
Culture-negative infection	5 (10.8)

a *Coagulase-negative Staphylococci*.

### Rotating Hinge Knee Arthroplasty Overall Survival

**Figure 2:**
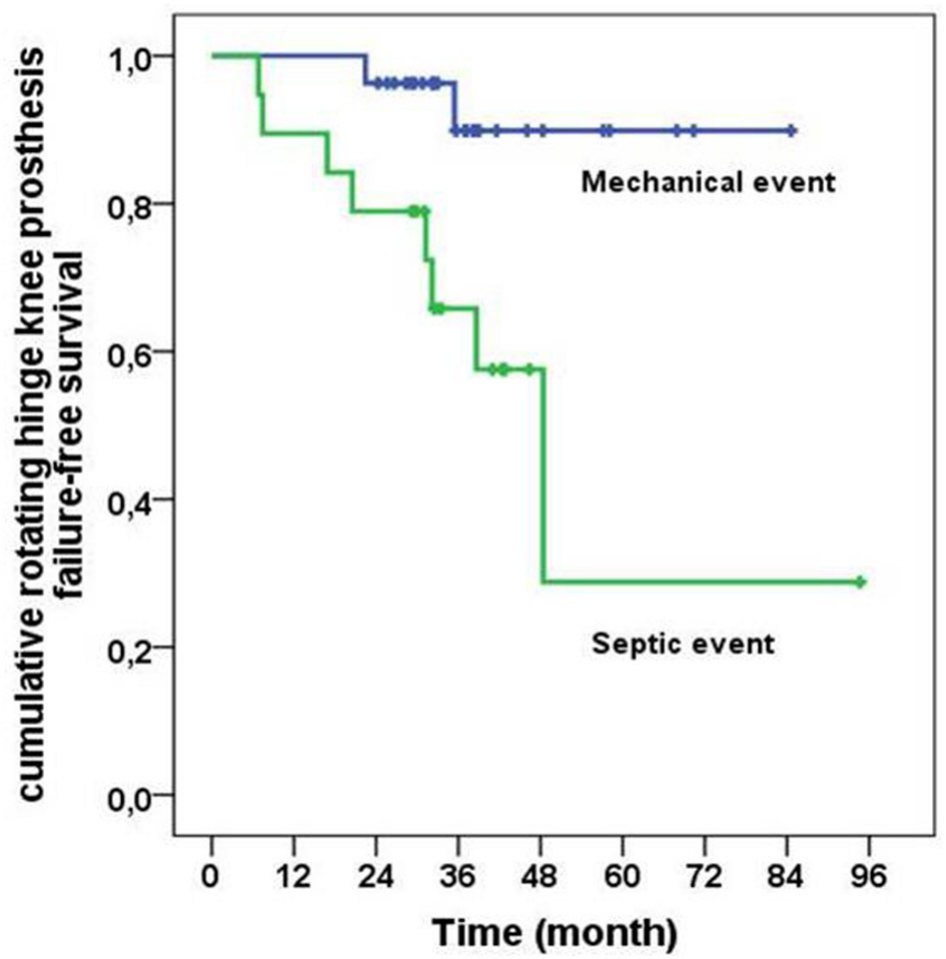
Cumulative probability of survival of rotating hinge knee prosthesis, depending on the cause of the explantation, mechanic vs. septic (log-rank = 0.09).

The 2-year overall survival rate was 89% but dropped to 65% after 7 years of follow-up. A significantly higher risk for implant removal due to septic causes compared to mechanical ones was observed (HR: 6.73; CI: 1.42–31.81; *p* = 0.016) (Figure 2). Out of the 10 implants removals, 8 were due to septic failure. Nineteen patients (44.1%) did not undergo any surgery following reimplantation.

### Mechanical Complications During the Follow-Up

Fifteen patients (32.6%) experienced at least one complication, detailed in Table 5. One patient underwent a one-stage revision of a TKA after mechanical loosening of the femoral component. Another patient underwent trans-femoral amputation for aseptic bipolar loosening. This was a relatively young patient who had already undergone several revision surgeries and requested a definitive solution.

**Table 5:** Mechanical complications (46 patients followed >2 years).

**Complications**	***n*** **(%)**
Spacer dislocation	2 (4.2)
Extensor apparatus complications	9 (19.6)
Peri-prosthetic fractures	4 (8.5)
Femur	1 (2.1)
Tibia	2 (4.3)
Patella	1 (2.1)
Neurologic complications (external popliteal sciatic nerve)	2 (4.3)
Aseptic loosening	3 (6.3)
Femur	1 (2.1)
Tibia	0 (0)
Patella	1 (2.1)
Bipolar loosening	1 (2.1)
Major stiffness (flexion <80°)	5 (10.9)
Algoneurodystrophy	1 (2.1)

### Septic Complications During the Follow-Up

Out of the 46 patients with >2 years of follow-up after reimplantation with a hinged TKA, 19 (41.3%) had at least one infectious event in their knee. The mean time of the infectious event was 16.4 months following the definitive surgery. Of note, five patients underwent an infectious event in the 3 months following reimplantation (among them, three were superinfections and two were persistent infections). The involved pathogen epidemiology of these infectious events is presented in Table 6. Most of them were superinfections (14/19) with new pathogen isolation, and none seemed to be of hematogenous in origin. Among them (i) six were resistant to cefazolin (the usual antimicrobial prophylaxis used at the time of reimplantation), including two multidrug-resistant (MDR) *Enterobacteriaceae*, two *P. aeruginosa*, one MDR *S. epidermidis*, and one *E. faecalis*; (ii) three were resistant to gentamicin (the usual antibiotic in the cement used to fix the hinged prosthesis), and three had a low level of resistance to gentamicin. Among the pathogens responsible for superinfections (i) cefazolin and gentamicin were both active in six of them but failed to prevent the superinfection; (ii) cefazolin and/or gentamicin were not active in eight of them, leading to reconsideration of the systemic and local antimicrobial prophylaxis. Out of these 19 patients (i) 10 were treated with Debridement Antibiotics and Implant Retention (DAIR), including four patients for whom iterative DAIR was performed; (ii) eight were treated with implant removal, among whom two had a new hinged TKA reimplanted, five underwent arthrodesis, and one with no reimplantation proposed (resection arthroplasty); and (iii) one patient had a transfemoral amputation.

**Table 6:** Epidemiology of pathogens involved in septic failures (19 patients).

**Pathogens**	***n*** **(%)**
Persistent infection	2 (10.5)
*Enterobacteriaceae*	2 (10.5)
*Streptococcus* spp.	2 (10.5)
Culture-negative infection	2 (10.5)
Superinfection	15 (78.9)
*Streptococci*	3 (15.8)
Enterobacteriaceae^*^	4 (21.1)
Staphylococci^*^	4 (21.1)
*P. aeruginosa*	2 (10.5)
*E. faecalis*	1 (5.2)
*P. multocida*	1 (5.2)

* *Including two multidrug-resistant (MDR) isolates*.

### Infectious Events Risk Factors

**Figure 3:**
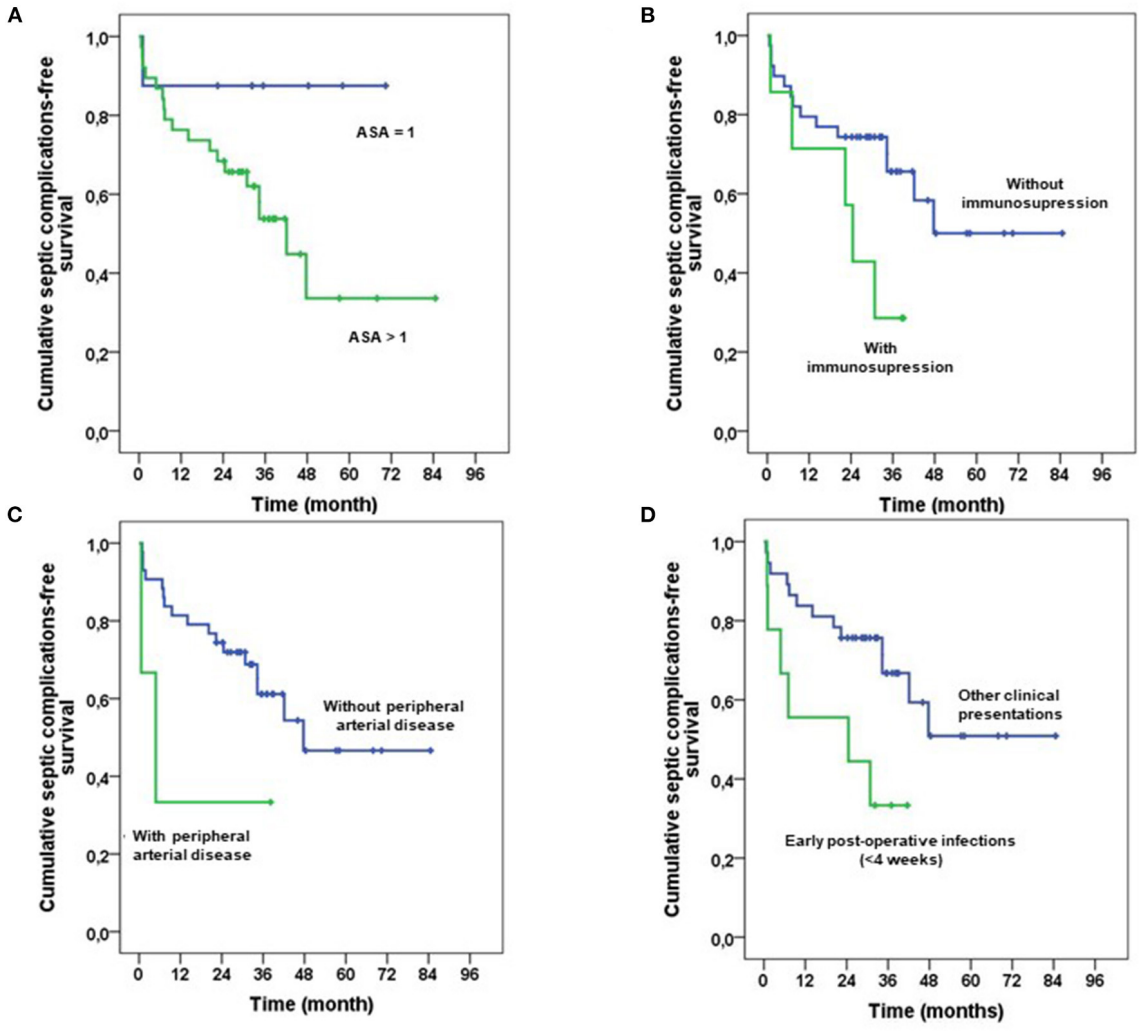
Cumulative probability for the infectious failure of rotating hinge knee prosthesis, depending on the ASA score (**A**; Log-Rank = 0.09), on the immunosuppression status (**B**; log-rank = 0.06), on the presence or not of peripheral arterial disease (**B**; log-rank = 0.10), and on type of infection depending on Tsukayama classification (**D**; log-rank = 0.02).

Evaluation of risk factors for septic failure revealed that age did not influence the outcome (Table 7). Patients with ASA score>1, immunosuppression, and with peripheral arterial diseases seemed to have a higher risk for septic failure (Table 7; Figures 3A–C). Patients with acute infection as initial clinical presentation were at higher risk, according to Tsukayama classification, in comparison with other patients (Table 7; Figure 3D). The variable “acute infection as initial clinical presentation according to Tsukayama classification” remained independently associated with septic failure in three different multivariate Cox models that, respectively, included age, ASA score>1, and peripheral arterial diseases but was not independent with the immunosuppressive status (Table 8). Among the nine patients with an acute infection as initial clinical presentation according to Tsukayama, four of them were immunosuppressed (4/9 vs. 3/37, *p* = 0.02 with Fisher test), and five of them experienced a superinfection. All of these cases were treated with a two-stage procedure.

**Table 7:** Univariate Cox analysis revealing risk factors for infectious failure.

	**Univariate analysis**		
	**HR^a^**	**95% CI^b^**	***p***
Age (per 10 years)	0.73	0.48–1.10	0.13
ASA>1	4.93	0.65–37.33	0.12
Immunosuppression	2.61	0.92–7.43	0.07
Peripheral arterial disease	3.28	0.74-14.44	0.12
Acute infection as initial clinical presentation according to Tsukayama	3.02	1.11–8.19	0.03
Acute infection as initial clinical presentation according to Zimmerli	4.11	0.91–18.5	0.07

a *Hazard ratio*.

b *95% CI*.

**Table 8:** Multivariate Cox analysis.

	**HR^a^**	**95% CI^b^**	***p***
**Multivariate Cox model n°1**			
Acute infection as initial clinical presentation according to Tsukayama	3.12	1.14–8.58	0.027
Age (per 10 years)	0.70	0.44–1.10	0.120
**Multivariate Cox model n°2**			
Acute infection as initial clinical presentation according to Tsukayama	2.97	0.10–8.50	0.032
ASA score >1	4.86	0.64–36.81	0.126
**Multivariate Cox model n°3**			
Acute infection as initial clinical presentation according to Tsukayama	2.46	0.83–7.26	0.104
Immunosuppression	1.895	0.61–5.89	0.269
**Multivariate Cox model n°4**			
Acute infection as initial clinical presentation according to Tsukayama	2.96	1.08–8.09	0.034
Peripheral arterial diseases	3.11	0.70–13.95	0.138

a *Hazard ratio*.

b *95% CI*.

### Functional Scores

Four patients could not undergo long leg x-rays because of an inability to fully weight bear. As a result, average “knee” IKS scores were calculated for 32 patients. The average IKS “knee” score was 70.5 points, CI 95% [63.9; 77.1] (*n* = 32 patients). The average IKS “function” score was 46.5 points, CI 95% [36.0; 57.0] (*n* = 36 patients). The average knee flexion was 88.7°, CI 95% [81.0; 96.5].

## Discussion

The main finding of this study is that the use of rotating hinged arthroplasty as a revision of a prosthetic-knee infection offers satisfactory functional outcomes following septic revision knee surgery but with a significant reinfection rate. The implications of these findings should encourage research studies toward alternative infection prevention pathways.

Data about rotating hinge knee arthroplasty survival are limited, especially for septic revision knee arthroplasty (5, 28–32) (Table 9). Disparities among survival rates could be explained by the heterogeneous distribution of hinged TKAs indications in the literature (9, 18, 33–37). Farid et al. (36) presented survival results for hinged TKAs in septic revisions. The survival rate (78.4%, mean follow-up ~5 years) was higher than that observed in this study (65%, mean follow-up ~7 years) in a cohort of 60 patients for whom a two-stage revision was performed. This may be explained by a younger population than ours (59.6 vs. 73.0 years), with significantly less comorbidities. Zahar et al. (38) studied the 10-year results of septic TKA revisions with rotating hinged prosthesis in 70 patients managed with a one-stage exchange. In this study, 93% of their patients were considered cured of prosthetic joint infection at 10 years. One explanation could be the wider indication of hinged TKAs in the study of Zahar than in our institution, where hinged implants were used only for severe prosthetic knee infections (5). Furthermore, this study only included patients for whom the pathogen was known before surgery and did not specify the distribution of acute or chronic infections, which may be a crucial element to interpret their results. Finally, the patients who did undergo a new surgical procedure after reimplantation (75% at 10 years, CI95% [60–87%]) were not systematically considered as a failure, and criteria for successful infection control in this study were defined as no clinical signs of infection, no further surgery with the diagnosis of periprosthetic joint infection (PJI), and no further positive cultures after the one-stage septic exchange (38).

**Table 9:** Literature review about septic revision managed with hinged prosthesis.

**Study (date)**	**Number of septic revisions with hinged prosthesis (Number of patients in the cohort)**	**Surgical strategy**	**Mean follow-up [min-max]**	**Type of implant**	**Survival**	**Functional outcomes**	**Post-operative complications**
Pradhan et al. (7)	23 (51)	2-stage	4 years [2-6]	Endo-Model®	np	Pre-operative HSS^a^ score:32 Post-operative HSS^a^ score: 70	- moderate pain: 3/23 - Amputation for septic recurrence: 1/23 - 6 plastic surgeries - persistent pain and stiffness: 1/23
Deehan et al. (33)	11 (72)	2-stage	10 years [3-18]	Howmedica Kinematic rotating hinge	90% at 5 years follow-up, across indications	Across indications: Knee Society Score 28–74	−18% (2/11) of reinfections following septic revision - Across indications: persistent pains (14%), extensor apparatus dysfunction (7%), Infection (7%), Peri-prosthetic fracture (4%)
Molenaers et al. (34)	29 (66)	2 stages	5 years [2-12]	Finn/OSS Biomet	92% at 5 and 10 years, across indications	KSS^b^ +27 points KSS^b^ pain + 12 points KSS^b^ function +20 points	−1 septic recurrence - Other septic revisions complications unspecified
Smith et al. (35)	46 (111)	Np	Np	- Kinematic 1 Stryker - Kinematic 2 Stryker - Duracon Total Knee System-Modular Rotating Hinge, Stryker - S-ROM Revision Hinge Knee, DePuy - Finn Hinge Knee Rotating Platform System Biomet	77% at 1-year follow-up, 52% at 5 years follow-up, across indications	Np	Across indications: - 63 % complications - 24% infection - 12% soft tissue complications (extensor apparatus and/or scar) - 7% aseptic loosening - 5% peri-prosthetic fracture
Shen et al. (9)	29 (94 hinged prosthesis, 381 non-hinged prosthesis)	Np	6 years [3-10]	Np		- Better functional outcomes of hinged TKA in patients with AORI^d^ type II bone loss in septic indication - Improved WOMAC^c^ score for hinged TKA in patients with AORI^d^ type III bone loss in septic indication	
Farid et al. (36)	60 (142)	2 stages	57 months [24-163]	OSS Biomet	78.4%	Np	−2-staged revision failure: 21.0.6% - Any cause failure: 26% - Reoperation: 38.5% - Aseptic loosening: 9.2% - Mechanical complications of the hinge: 6.1% - Extensor apparatus complications: 6.1% - Peri-prosthetic fracture 6.1% - Femoral stem fracture: 7.7%
Cottino et al. (37)	144 (408)	2 stages	48 months [24-144]	- Howmedica modular rotating hinge - NexGen RH Knee Zimmer - S-ROM Noiles rotating Hinge Depuy - Finn Rotating Hinge Biomet	Across indications: - 84.5% at 5 years follow-up - 71.3% at 10 years follow-up	Across indication: KSS^b^: from 51 to 81 KSS^b^ function: from 26 to 36	Across indications: - Infection (11%) - Delayed wound healing (3%) - Stiffness (2.5%) - Aseptic loosening (2.5%) - Superficial infection (1.2%)

HSS^a^ *score de l'Hospital Special Surgery*.

KSS^b^ *Knee Society Score*.

**WOMAC^c^:** *score Western Ontario McMaster*.

**AORI^d^:** *Anderson Orthopaedic Research Institute*.

*Np, no precisions*.

Considering functional outcomes, previous studies have not used the same evaluation scores and often with heterogenous indications, making interpretation difficult (Table 9). In this study, IKS “knee” scores seemed lower than those found in the literature (33, 37). However, our IKS “function” scores were in line with the literature (13, 14) or slightly more favorable (8, 37). Globally, functional scores are worse in reported series including hinged TKAs used in septic revisions (8, 13, 14, 33, 37) than in cohorts only studying non-septic indications (first-line arthroplasties or mechanical revisions) (33, 34). Nevertheless, the mean range of flexion found in our study was slightly better than that observed in the study of Zahar et al. (32) (respectively, 88.7° vs. 76°).

In our study, patients with chronic knee PJI requiring revision with rotating hinge knee arthroplasty experienced a high rate of septic recurrence. We found that acute infection as initial clinical presentation according to Tsukayama was a significant risk of septic failure defined by the need for subsequent surgery such as DAIR or implant removal due to clinical signs of infection occurrence. It is unclear why patients with an acute presentation should be more at risk of septic failure, especially as the different mechanisms of persistence such as biofilm are usually developed by the bacteria during chronic infections. It is possible that acute presentation could be potentially associated with a high bacterial inoculum or could be associated with more inflammation among periprosthetic soft tissue that may facilitate bacterial superinfection. Most septic failures were due to bacterial superinfections, probably acquired during reimplantation, despite following the WHO guidelines for the prevention of infection, such as the use of systemic cefazolin and the use of gentamicin-loaded cement for the prosthesis fixation as prevention (23). Checking the antibiogram of each pathogen responsible for superinfection, we found that cefazolin and/or gentamicin were not active in 8 out of the 19 superinfections, leading to reconsider systemic and/or local antimicrobial prophylaxis pathways. We found that patients with ASA score >1, with immunosuppression, or with arterial vascular diseases were at higher risk. Thus, these patients crucially need additional innovative approaches to reduce the rate of superinfection. A more efficient systemic antimicrobial prophylaxis and the use of particular antibiotics-loaded cement for prosthesis fixation could be alternative options. The first option would be using a beta-lactam with a wider spectrum of activity than that of cefazolin. The only one that could target all the involved pathogens in superinfections, except for multi-drug-resistan (MDR) *Staphylococci*, would be imipenem. Unfortunately, it is not possible to use imipenem as systemic prophylaxis, since it is considered as a last resort antibiotic that must be kept for MDR severe infections (39). The second option would be adding systemic gentamicin to cefazolin to increase the spectrum of activity on *Enterobacteriaceae, P. aeruginosa*, and *E. faecalis*. Of note, two MDR *Enterobacteriaceae* responsible for superinfection in our study were gentamicin-resistant, and all of our patients received gentamicin as local antimicrobial prophylaxis in the cement used to fix the prosthesis. The final option would be a combination of antimicrobials in the cement used for reimplantation. For that purpose, it is important to use commercial cements that guarantee the mechanical strength of the fixation (39). Manually adding antibiotics into the cement during its preparation is technically feasible for a spacer but is controversial when the cement has only been approved and designed to fix prosthesis (39). Few antibiotic-loaded cements releasing a combination of antimicrobials are available on the market. Gentamicin- and clindamycin-loaded poly-methyl methacrylate (PMMA) cement is available in Europe, but we do not consider it as useful for our patients, even if the dose of gentamicin is higher compared to the one we used, since there is no added value of the clindamycin in terms of the spectrum of activity. An aminoglycoside (tobramycin or gentamicin) could be combined with vancomycin in a PMMA spacer: tobramycin- and vancomycin-cement are available in the US (40), and gentamicin- and vancomycin-cement are available in Europe (41). These cements are interesting as their spectra of activity cover aminoglycoside-sensitive *Enterobacteriaceae, E. faecalis*, and most of the *Staphylococci*, including MDR *staphylococci*. In our study, using this kind of cement during reimplantation would have had an activity on all pathogens responsible for superinfections, except on the two MDR *Enterobacteriaceae* that were also aminoglycoside-resistant. An alternative could have been to use intrawound vancomycin combined with gentamicin PMMA cement. In a recent study that included patients with primary arthroplasty, intrawound vancomycin seems to decrease early periprosthetic joint infection (42). But with this route of application, the local release of vancomycin is probably limited in time, unlike cements that last several days (41). Finally, an additional measure would be to propose *S. aureus* decolonization before reimplantation (43), but only 1 patient out of the 19 developed post-operative *S. aureus* superinfection.

Our study had several limitations. First, there was an obvious selection bias since all patients were managed at the Lyon University hospitals. This also explained most two-stage procedures, which remain the gold standard (44–47). Then, although the number of one-stage-managed patients was low (*n* = 3), this probably heterogenized our study, and we could not establish two comparative groups (one-stage vs. two-stage). In the literature, the meta-analysis of Kunudsor et al. (48) found similar reinfection rates between one- and two-stage exchanges [7.6% CI 95% [3.4–13.1], *p* < 0.001 vs. 8.8% CI 95% [7.2–10.6], *p* < 0.001]. Functional scores were similar between the two groups (IKS score and range of motion). Even if the sample size was low in our study, all patients requiring septic revision were managed in the same way at the stage of rotating hinged prosthesis reimplantation. Last, despite the low sample sizes, we recorded essential signals (high rate of superinfection, particularly in comorbid patients) that must be considered to implement innovative preventive measures in such a population.

## Conclusions

Hinged prostheses in septic revisions of TKAs are a therapeutic alternative with contrasting results. When successful, they offer satisfying functional outcomes and good survival results in the short and medium terms; however, complications are frequent, specifically infectious events. Efforts have to be made in the prevention of superinfections, especially for patients with immunosuppression and peripheral arterial diseases, since the risk of infections after TKA revision with hinged prosthesis is high. These patients require optimal antimicrobial systemic prophylaxis and innovative approaches to reduce the rate of superinfection. More research studies are needed to further evaluate optimal antimicrobial prophylaxis and to identify innovative approaches to reduce the rate of superinfection.

## Data Availability Statement

The original contributions presented in the study are included in the article/supplementary material, further inquiries can be directed to the corresponding author.

## Ethics Statement

The studies involving human participants were reviewed and approved by Hospices Civils de Lyon Ethic Committee. Written informed consent from the participants' legal guardian/next of kin was not required to participate in this study in accordance with the national legislation and the institutional requirements.

## Author Contributions

SL, TF, and FB-S contributed conception and design of the study. FB-S organized the database and wrote the first draft of the manuscript. TF and MC performed the statistical analysis. TF wrote sections of the manuscript. MB-L translated the manuscript. All authors contributed to manuscript revision, read, and approved the submitted version.

## Conflict of Interest

The authors declare that the research was conducted in the absence of any commercial or financial relationships that could be construed as a potential conflict of interest.

## Publisher's Note

All claims expressed in this article are solely those of the authors and do not necessarily represent those of their affiliated organizations, or those of the publisher, the editors and the reviewers. Any product that may be evaluated in this article, or claim that may be made by its manufacturer, is not guaranteed or endorsed by the publisher.
